# A common CD55 rs2564978 variant is associated with the susceptibility of non-small cell lung cancer

**DOI:** 10.18632/oncotarget.14053

**Published:** 2016-12-20

**Authors:** Yanyan Zhang, Zhi Zhang, Lei Cao, Jia Lin, Zhenbang Yang, Xuemei Zhang

**Affiliations:** ^1^ Institute of Molecular Genetics, College of Life Science, North China University of Science and Technology, Tangshan, China; ^2^ Institute of Epidemiology, School of Public Health, North China University of Science and Technology, Tangshan, China; ^3^ Affiliated Tangshan Gongren Hospital, North China University of Science and Technology, Tangshan, China

**Keywords:** CD55, single nucleotide polymorphism, complement, lung cancer, susceptibility

## Abstract

CD55, as one of key membrane-bound complement-regulatory proteins (mCRPs), is crucial for the progression of various cancers. This study aims to investigate the role of CD55 variants in the development of non-small cell lung cancer (NSCLC). A case-control study, including 706 lung cancer cases and 706 health controls, was conducted in a Chinese population. The odds ratio (*OR*) and 95% confidence interval (*95% CI*) were estimated by unconditional logistic regression. We found that significantly higher lung cancer risk was linked with *CD55* rs2564978 CC genotype (*OR* = 1.52, *95% CI* = 1.11−2.07) or CT genotypes (*OR* = 1.34, *95% CI* = 1.05−1.71), compared to the TT genotype. Stratified analysis showed that rs2564978 CC was associated with NSCLC risk among males (*OR* = 1.69, *95% CI* = 1.14−2.49) and older subjects (*OR* = 1.75, *95% CI* = 1.08−2.82). When stratified by smoking status, the risk effect of rs2564978 CC was more evident among smokers (*OR* = 2.01, *95% CI* = 1.18−3.43) than non-smokers (*OR* = 1.30, *95% CI* = 0.88−1.90). We also conducted the stratified analysis by NSCLC histological types and found that *CD55* rs2564978 CC increased the risk of adenocarcinoma with OR (*95% CI*) of 1.35 (1.01−1.80). The reporter gene expression driven by rs2564978T-containing CD55 promoter was respectively 1.48-fold, 1.96-fold and 1.93-fold higher than those driven by the rs2564978C-containing CD55 promoter in A549, NCI-H2030 and NCI-H23 cells (*P* = 0.045, 0.010 and < 0.001). These findings indicate that *CD55* rs2564978 polymorphism may contribute to an increased risk of NSCLC in Chinese population.

## INTRODUCTION

Lung cancer, as one of the most common cancers worldwide, is the first-leading cause of cancer-related death in China [1, 2]. Lung cancer has two main types: non-small cell lung cancer (NSCLC) and small cell lung cancer (SCLC). The vast majority (80%) of cases of lung cancer are NSCLC [3, 4]. The development of NSCLC has been taken into account as an environmental and genetic event. Several genome-wide association studies (GWAS) have identified a number of genetic signatures, which correlate with cancer development [5, 6].

Innate complement control system is part of fundamental innate immune defense mechanisms involved infighting against cancer by exerting immune-surveillance [7–9]. *CD55* (decay accelerating factor, *DAF*) is a glycosylphosphatidylinositol (GPI)-anchored membrane inhibitor of complement. DAF inhibits complement activation by interfering with C3/C5 convertases in both classical and alternative pathways [10, 11]. Physiologically, the expression of *CD55* protects host cells from the damage of pathogenic microorganisms. The expression *CD55* in gastric cancer, colon and breast cancer was significantly greater than in those non-cancer tissues [12–16]. However, studies also demonstrated a down-regulated expression of *CD55* in ovarian cancer and lung cancer tissues [17, 18]. In Higuchi's study, *CD55* was recognized as a novel PNA-binding protein in the human lung and the down-regulation of *CD55* was associated with pathology of primary NSCLC [17].

Based on the possible function of *CD55* in cancer development, we hypothesized that *CD55* genetic variants contribute to the risk of developing NSCLC. In this present study, we conduct a case-control study to investigate the association of *DAF* genetic variant with the susceptibility of NSCLC. Furthermore, we provide evidence to suggest the effect of this polymorphism on the promoter activity of *DAF* gene.

## RESULTS

### Subject characteristics

The principal demographic and clinical characteristics of the participants summarized in Table 1. No statistically significant differences were observed in the distributions of age and gender between the cases and controls. It was found that more smokers were present among lung cancer cases than health controls (44.8% vs. 28.3%, *P* < 0.001). However, there was no significant difference in light and heavy smokers between cases and controls. Among lung cancer cases, 57.2% were classified as adenocarcinoma, 38.7% as squamous-cell carcinoma, and 4.1% as other types, including adenosquamous carcinoma (*n* = 15), large cell carcinoma (*n* = 5), and alveolar carcinoma (*n* = 9).

**Table 1:** Distributions of select characteristics by cases and controls subjects

Variables	Case (*n* = 706)		Controls (*n* = 706)		*P* value^a^
	No	(%)	No	(%)	
Sex					0.87
Male	471	66.7	474	67.1	
Female	235	33.3	232	32.9	
Age					1.00
≤ 50	125	17.7	125	17.7	
51–60	265	37.5	265	37.5	
61–70	226	32.0	226	32.0	
> 70	90	12.7	90	12.7	
Smoking status					0.00
Non-smoker	390	55.2	506	71.7	
Smoker	316	44.8	200	28.3	
Pack-year smoked					0.74
≤ 30	188	59.5	116	58.0	
> 30	128	40.5	84	42.0	
Histological types					
Adenocarcinoma	404	57.2			
Squamous cell	273	38.7			
Other carcinomas	29	4.1			

^a^Two-sided χ^2^ test.

### Association of *CD55* polymorphism and the risk of lung cancer

Distribution of *CD55* rs2564978 genotypes was showed in Table 2. Genotype distribution of *CD55* rs2564978 in controls conformed to the Hardy-Weinberg equilibrium (HWE) (*P* = 0.81). The genotype frequencies of *CD55* rs2564978 (27.8% for TT, 51.3% for CT and 21.0% for CC) among case patients were significantly different from those (34.3% for TT, 48.2% for CT and 17.6% for CC) among health controls (χ^2^ = 7.638, *P* = 0.022). Under unconditional logistic regression model, significantly higher risk of lung cancer was presented in individuals with rs2564978 CC genotype (*OR* = 1.52, *95% CI* = 1.11–2.07) or CT genotypes (*OR* = 1.34, *95% CI* = 1.05–1.71), compared with those with TT genotype.

**Table 2:** Genotype frequencies of CD55 rs2564978 and their association with NSCLC

Genotypes	Controls (*n* = 706)		Cases (*n* = 706)		OR (95% CI)^a^	*P* value
TT	242	34.3	196	27.8		
CT	340	48.2	362	51.3	1.34(1.05-1.71)	0.021
CC	124	17.6	148	21.0	1.52(1.11-2.07)	0.008

^a^Data were calculated by unconditional logistic regression and adjusted for sex, age and smoking status.

### Stratification analysis of the *CD55* polymorphism and the risk of lung cancer

We then performed stratification analysis to evaluate the association of *CD55* rs2564978 genotypes with lung cancer (Table 3). Compared with the rs2564978 TT genotype, we found a significantly increased risk of lung cancer was association with CC genotype among males (*OR* = 1.69, *95% CI* = 1.14–2.49), but not among females (*OR* = 1.14, *95% CI* = 0.67–1.92). In stratified analysis by age, the older subjects (age > 60) with rs2564978 CC genotype had an increased risk of lung cancer with *OR* (*95% CI*) of 1.75 (1.08–2.82), but younger subjects didn't (*OR* = 1.37, *95% CI* = 0.91–2.06). We then investigated whether a gene-smoking interaction existed between the genotypes and smoking. We concluded that the risk effect of rs2564978 CC genotype was more evident among smokers (*OR* = 2.01, *95% CI* = 1.18–3.43), but not among non-smokers (*OR* = 1.30, *95% CI* = 0.88–1.90). However, we didn't find that *CD55* rs2564978 T > C variant effected on the risk of lung cancer when stratified by the smoking intensity. We also conducted the stratified analysis by histological types of NSCLC and found that *CD55* rs2564978 CC increased the risk of adenocarcinoma with *OR* (*95% CI*) of 1.35 (1.01–1.80). However, we didn't find *CD55* rs2564978 polymorphism effect on the risk of squamous-cell carcinoma (CC versus TT, OR = 0.06, 95% CI = 0.99–2.30) and other types (CC versus TT, OR = 2.36, 95% CI = 0.70–7.95).

**Table 3:** Stratified analysis between CD55 rs2564978 genotypes and NSCLC risk

Variables	Genotypes (Cases/Controls)			CC/TT model OR (95% CI)^a^	*P* value
Sex					
Male	127/178	252/216	92/80	1.69 (1.14–2.49)	0.008
Female	69/64	110/124	56/44	1.14 (0.67–1.92)	0.624
Age					
≤ 60	108/132	197/183	85/75	1.37 (0.91–2.06)	0.134
> 60	88/110	165/157	63/49	1.75 (1.08–2.82)	0.022
Smoking status					
Non-smoker	110/160	194/252	86/94	1.30 (0.88–1.90)	0.184
Smoker	86/82	168/88	62/30	2.01 (1.18–3.43)	0.010
Pack-year of smoking					
≤ 30	47/44	104/54	37/18	1.95 (0.96–3.94)	0.063
> 30	39/38	64/34	25/12	2.00 (0.87–4.57)	0.102
Histological types					
Adenocarcinomas	110/242	210/340	84/124	1.35 (1.01–1.80)	0.042
Squamous cell	81/242	134/340	58/124	0.06 (0.99–2.30)	0.057
Other carcinomas^b^	5/242	18/340	6/124	2.36 (0.70–7.95)	2.361

^a^Data were calculated by unconditional logistic regression and adjusted for sex, age and smoking status.

^b^Other carcinomas: adenosquamous carcinoma (*n* = 15), large cell carcinoma (*n* = 5), alveolar carcinoma (*n* = 9).

### Functional role for *CD55* polymorphism by luciferase reporter assays

**Figure 1:**
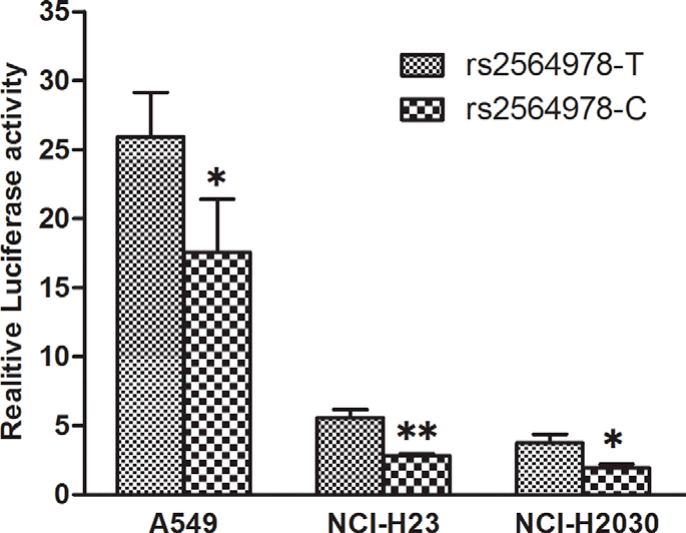
Effect of the CD55 rs2564978 polymorphism on the CD55 promoter activity Transient reporter gene expression assays with constructs containing the *CD55* rs2564978 C or T allele, which was inserted at upstream of the luciferase reporter gene in the pGL3-Basic plasmid. The two constructs were transfected into the A549, NCI-H2030 and NCI-H23 cells, respectively. All of the constructs were co-transfected with pRL-SV40 to standardize the transfection efficiency. Data shown are the means ± SD from 3 independent transfection experiments, each performed in triplicate. *P < 0.05 and **P < 0.01 compared with the pGL3-rs2564978T construct.

Having shown that *CD55* rs2564978 polymorphism effect on the risk of non small-cell lung cancer, we then conduct a dual-luciferase reporter gene assay to assess the effect of *CD55* rs2564978 polymorphism on transcriptional activity of the gene. Two different pGL3-CD55 promoter luciferase report constructs, which including the *CD55* rs2564978 T or C allele (pGL3-rs2564978T and pGL3-rs2564978C, respectively), were generated. These constructors were used to transiently transfect A549, NCI-H2030 and NCI-H23 lung cancer cells. As shown in Figure 1, the reporter gene expression driven by rs2564978T-containing CD55 promoter were 1.48-fold, 1.96-fold and 1.93-fold higher than those with rs2564978C-containing CD55 promoter in A549, NCI-H2030 and NCI-H23 cells (*P* = 0.045, *P* = 0.010 and *P* < 0.001, respectively).

## DISCUSSION

Currently, it is unclear if the polymorphism located in the promoter *CD55* is associated with the risk of developing lung cancer. In the present study, we found that the *CD55* rs2564978 T > C variant significantly decreased the transcriptional activity in lung cancer cells and contributed to an increased risk of NSCLC.

Human immune system included two distinct subsystems, innate immune and adaptive immune. Complement system served as a functional bridge between innate and adaptive immune responses [19]. As one of key membrane complement regulatory proteins (mCRPs), *CD55* activates T and B cells by binding to *CD97*, which is expressed on macrophages, granulocytes [12]. *CD55* also inhibits C3 and C5 activation and thereby limits complement-dependent cell cytotoxicity (CDC) [10, 13]. Studies have demonstrated a down-regulation of *CD55* in NSCLC cells [17]. Our present results supported that the *CD55* rs2564978 CC genotype was linked to an increased risk of NSCLC. This finding is similar with our previous result from gastric cancer case-control study [20]. This population data can be explained by our luciferase reporter gene assay. We found that C allele of rs2564978 was associated with reduced expression of *CD55* compared with the T allele in lung cancer cells. However, in human bronchial epithelial cells, Zhou's luciferase assay showed that *CD55* promoter with rs2564978 T allele exhibited significantly lower transcriptional activity than that with C allele [21]. These may illustrate the different role of *CD55* in normal cells and cancer cells.

Cigarette smoking is the most demonstrated risk factor for the development of lung cancer [22, 23]. Thus, we analyzed the role of *CD55* polymorphism in the development of lung cancer stratified by smoking status. Our results suggested that *CD55* rs2564978 variant was related to lung cancer among smokers, but not among non-smokers. Tobacco could induce chronic inflammation in lung microenvironment and modify host responses to exogenous antigens [24, 25]. In the process of inflammatory response, complement activation plays an important role. It has been reported that cigarette smoke induced the cleavage of C3 and C5 and further led to activation of the alternative complement pathway [26, 27]. As a key regulator of C3 and C5, the effect of *CD55* genetic variant to the risk of lung cancer might be altered by tobacco smoke.

In conclusion, our results indicated that rs2564978 C allele decreased the transcriptional activity of *CD55* and was associated with the susceptibility of NSCLC in a Chinese population.

## MATERIALS AND METHODS

### *CD55* SNP selection

Based on the Han Chinese in Beijing (CHB) population data from Ensembl, we selected the SNPs located in the promoter region of *CD55*, and the minor allele frequency (MAF) in HapMap was greater than 5%. Web-based Alibaba 2.1 ( http://www.gene-regulation.com/pub/programs/alibaba2/) was used for predicting transcription factor binding site. As a result, only one SNP in the promoter of *CD55* was selected for further analysis.

### Study population

This is a continuous case-control study involved 706 patients with lung cancer and 706 healthy controls [28, 29]. All subjects were genetically unrelated Han Chinese. All case subjects were newly diagnosed and histologically confirmed from Tangshan Gongren Hospital (Tangshan, China) since 2008. Health controls without history of any cancer were recruited from health checkup examinations and matched to patients by frequency according to age (± 5 years) and gender. This study was approved by the Institutional Review Board of North China University of Science and Technology. Informed consent was obtained from each subject before participation in this study. Epidemiology data were collected by interview. After interview, 2 ml blood samples were collected from each participant and for further laboratory analysis.

### *CD55* genotyping

Genomic DNA was extracted from peripheral blood samples using the TIANamp Blood DNA Kit (TIANGEN, Beijing, China). The genotype of *CD55* rs2564978 T > C polymorphism was determined by polymerase chain reaction - restriction fragment length polymorphism (PCR-RFLP). A 101-bp DNA fragment containing *CD55* rs2564978 site was amplified with the primer pairs of CD55F(5′-ATGAACAATGTTCACT CCCTACTGTGGTA-3′) and CD55R(5′-TAAGGAGGA AGGGCGTCATC-3′). PCR was performed in a 5 μl reaction mixtures containing 10ng of DNA, 0.1 μM of each primer and 1 × EsTaq PCR MasterMix (CWBIO, Beijing, China). The PCR profile consisted of an initial melting step of 3minutes at 94°C, followed by 30 cycles of 40 seconds at 94°C, 30 seconds at 61.5°C, 25 seconds at 72°C, and a final elongation step of 72°C for 3 minutes. PCR products were digested by Rsa *I* (New England Biolabs, Hitchin, UK). Genotyping was performed without knowledge of the case/control status. A 10% random sample was tested in duplicate by different persons, and all results were 100% concordant.

### Construction of reporter plasmids

To verify whether the identified CD55 promoter polymorphism effects on the transcriptional activity of *CD55*, we constructed a reporter plasmid containing 1975bp fragment between -1438 bp to 526bp of *CD55* promoter. The primers used for amplifying this *CD55* promoter were 5′-GGGGTACCCCTCTCTATGAAGAAGGGCA-3′ and 5′-CCCAAGCTTGGGGACGGCGGGAACCACGAC-3′, in which the underlined bases represented Kpn*I* and Hind *III* (NEB, MA, USA) cutting site. The PCR product was double digested with Kpn*I* and Hind *III* and then inserted into the upstream of firefly luciferase reporter gene of the pGL3-basic (Promega, Madison, USA). The resulting construct was designated as pGL3-rs2564978C or pGL3-rs2564978T according to sequence result. We then use this rs2564978 C (or T)-containing vector to get another rs2564978 T (or C)-containing one by site-specific mutagenesis. Before cell transfection, the sequence of each construct was confirmed by direct sequencing.

### Cell culture, transfection and luciferase assay

Human lung cancer cells (A549, NCI-H2030 and NCI-H23) were supplied from Cobioer Biosciences (Cobioer, Nanjing, China). All cells were grown in RPMI 1640 medium supplemented with 10% fetal bovine serum (FBS) (GIBCO, NY, USA) and 1% of penicillin and streptomycin in a humidified environment at 37^°^C with 5% CO_2_. For transient transfection, 2 × 10^5^ cells were seeded in 24-well culture plates to 60%–70% confluence. A549, NCI-H23 and NCI-2030 cells were transfected with 1ug firefly luciferase reporter plasmid by Lipofectamine 2000 (Invitrogen, CA, USA). Meanwhile, all plasmids were co-transfected with 10ng of pRL-SV40 plasmid (Promega, Madison, USA), which contained the Renilla luciferase gene as an internal control. The pGL3-Basic vector co-transfected with pRL-SV40 plasmid served as a control. Luciferase activity was determined using Luciferase reporter assay kit (Promega, Madison, USA) and measured in GloMax 20/20 luminometer (Promega, Madison, USA). For each plasmid construct, three independent transfection experiments were carried out, and each luciferase assay was performed in triplicate.

### Statistical analysis

The differences in demographic variables and the distribution of genotype between patients and controls were tested by χ^2^ test. Hardy-Weinberg equilibrium (HWE) for *CD55* rs2564978 C > T polymorphism in controls was evaluated using Pearson goodness-of-fit χ^2^ test. Unconditional logistic regression was used to analyze the associations of *CD55* rs2564978 variant with the risk of lung cancer by odds ratio (*OR*) and their 95% confidence interval (*95% CI*) with adjustments for age, gender, and pack-year of smoking. Light and heavy smokers were categorized by using the 50th percentile pack-year (cigarettes per day/20) × (years smoked), with the value of the controls as the cutoff points (i.e., ≤ 30 and > 30 pack-years). Luciferase activity data were presented as mean ± standard deviation (SD) and tested by t test. All statistical analyses were performed using the SPSS version 17.0 (SPSS Inc, Chicago, IL). A *P* value of < 0.05 was regarded as statistically significant.
